# Paquinimod prevents development of diabetes in the non-obese diabetic (NOD) mouse

**DOI:** 10.1371/journal.pone.0196598

**Published:** 2018-05-09

**Authors:** Sahar Tahvili, Marie Törngren, Dan Holmberg, Tomas Leanderson, Fredrik Ivars

**Affiliations:** 1 Immunology group, Section for Immunology, Department of Experimental Medical Science, Lund University, Lund, Sweden; 2 Active Biotech AB, Lund, Sweden; Children's Hospital Boston, UNITED STATES

## Abstract

Quinoline-3-carboxamides (Q compounds) are immunomodulatory compounds that have shown efficacy both in autoimmune disease and cancer. We have in here investigated the impact of one such compound, paquinimod, on the development of diabetes in the NOD mouse model for type I diabetes (T1D). In cohorts of NOD mice treated with paquinimod between weeks 10 to 20 of age and followed up until 40 weeks of age, we observed dose-dependent reduction in incidence of disease as well as delayed onset of disease. Further, in contrast to untreated controls, the majority of NOD mice treated from 15 weeks of age did not develop diabetes at 30 weeks of age. Importantly, these mice displayed significantly less insulitis, which correlated with selectively reduced number of splenic macrophages and splenic Ly6C^hi^ inflammatory monocytes at end point as compared to untreated controls. Collectively, these results demonstrate that paquinimod treatment can significantly inhibit progression of insulitis to T1D in the NOD mouse. We propose that the effect of paquinimod on disease progression may be related to the reduced number of these myeloid cell populations. Our finding also indicates that this compound could be a candidate for clinical development towards diabetes therapy in humans.

## Introduction

Type 1 Diabetes (T1D) is an autoimmune disorder that causes severe loss of pancreatic β-cells and insulin production [1]. T cells are key mediators of this process but understanding of the mechanisms that underlie T cell dysregulation in humans with T1D is limited [2]. The most prominent therapy offered to T1D patients is exogenous insulin administration. This treatment, however remains suboptimal and fails to prevent severe complications of the disease.

The non-obese diabetic (NOD) mouse model is a good model for T1D and while developing a more severe insulitis than commonly seen in the human disease, displays several characteristics common to human T1D [3–5]. The development of T1D in the NOD mouse is spontaneous and highly T cell-dependent. Already at 3–4 weeks of age there is detectable insulitis in these mice, causing selective cell death of the insulin producing β-cells in the islets of Langerhans as the disease progresses [3–5]. Female NOD mice display severe insulitis at about 15 weeks of age and develop glycosuria at around 15–30 weeks of age.

The initial phase of insulitis involves early presence of macrophages and dendritic cells [6–9]. This is followed by recruitment of self-antigen specific CD4 and CD8 T cells (reviewed in [10, 11]) and cell transfer experiments demonstrated that both CD4 and CD8 T cells participate in the β-cells destruction process [12–14]. The autoimmune T cell response is directed to self-antigens expressed by the β-cells (reviewed in [15]) and the initiation of the response involves presentation of islet antigens by islet-derived DCs in draining pancreatic lymph nodes [16–19]. Further, DCs were found to be critical for maintaining the T cell response [20]. It has been reported that DCs in NOD mice produce elevated level of IL-12 and have elevated T cell stimulatory capacity [21, 22]. In addition to DCs and macrophages, other cells such as neutrophils, plasmacytoid DCs [6, 23], NK cells [24, 25] and B cells [26, 27] have also been shown to be involved in the disease process.

A large variety of therapeutic approaches have been attempted in the NOD mouse model and shown efficacy on progression on T1D and even recovery of β-cell activity (reviewed in [28–30]). Some of these pre-clinical studies involved antibody-based therapies targeting T cells [31–33] or B cells [34, 35]. These studies led to clinical trials that have also shown some beneficial effects on T1DM in patients (reviewed in [29, 36, 37].

Quinoline-3-carboxamides (Q compounds) are immunomodulatory compounds [38–40] that have shown efficacy in several different experimental models of human inflammatory disease [40–44] and cancer (reviewed in [45, 46]). One such compound, paquinimod, was shown to bind the S100A9 protein and prevent its interaction with TLR4 [47]. S100A9 is an intracellular calcium-binding protein that is released and detected in circulation in various inflammatory conditions [48–50]. Since the binding of S100A9 to TLR4 stimulates a pro-inflammatory cytokine-response in monocytes [51–53], the finding that paquinimod blocks that binding provided one candidate mechanism for the efficacy of the compound in inflammatory disease. We have in our previous work investigated the efficacy of the Q compound paquinimod on disease development in experimental autoimmune encephalomyelitis (EAE), a model of T cell-mediated autoimmunity [43] and in peritonitis, a model of acute inflammation [42]. In both of these studies [42, 43] as well as other studies from our laboratory [54, 55], we could show selective effects of Q compounds on inflammatory monocytes [42, 43, 54], eosinophils [42, 54] and myeloid (CD11b^+^) dendritic cells [55]. Importantly, in the EAE model a selective reduction of inflammatory monocytes correlated with a reduced in vivo T cell response [43]. Several other laboratories have also reported that treatment with the Q compound laquinimod, which is structurally similar to paquinimod, could reduce the proportion of disease-causing Th1 and Th17 cells in the EAE model [40, 56–59], and that this modulation may be mediated via effects on myeloid antigen-presenting cells and not due to direct effects on the T cells themselves [56, 57].

Administration of exogenous insulin is currently the most common treatment for human T1D. The disease progresses in treated individuals and they display various disease-associated complications. There is therefore demand for novel treatments that would inhibit progression of the disease and ideally would also allow for regeneration of β-cell mass in the pancreatic islets. Stem cell-based strategies provide examples of such novel treatments [60, 61]. Since paquinimod in previous studies had shown efficacy in other T cell-dependent disease models, we hypothesized that paquinimod treatment might also have beneficial effects on the development of T cell-dependent T1D in the NOD mouse. To address this hypothesis, we have in this report treated NOD mice with paquinimod and investigated the efficacy of treatment on the development of insulitis and T1D. In line with our hypothesis, we found significantly reduced incidence and delayed onset of diabetes in the treated mice. Further, the reduced incidence of disease correlated with amelioration of insulitis. Analyses of cells isolated from spleen and pancreatic lymph nodes (panLN) revealed selective reduction of subpopulations of myeloid cells in the treated mice. In particular, the number Ly6C^hi^ inflammatory monocytes were reduced in spleen. Collectively our results demonstrate that the immunomodulatory compound paquinimod is a potent inhibitor of insulitis and diabetes development in the NOD mouse.

## Materials and methods

### Mice and treatment

Female NOD/MrkTac mice were purchased from Taconic (USA). All animal experiments were performed with the permit of the local committee for the ethics of animal experiments of Malmö and Lund (permits M281-01 and M42-14). Mice were exposed to increasing concentration of CO_2_ and upon loss of consciousness euthanized by cervical dislocation. To investigate the effect of the Q-compound paquinimod on development of glycosuria and insulitis, mice were treated with the compound dissolved in drinking water at different concentrations corresponding to daily doses of about 0.04, 0.2, 1, and 5 mg/kg body weight/day). The mice were treated with paquinimod starting from either 10 or 15 weeks of age. The duration of treatment varied from 5 to 23 weeks in the different experiments performed. Paquinimod was obtained from Active Biotech, Lund, Sweden.

### Diabetes incidence, insulitis scoring, and histological analysis

Mice were monitored for diabetes incidence by weekly measurement of glucose levels in urine (Keto-Diaburtest 5000 kit, Roche). Mice were considered diabetic when glucose level in urine was more than 13 mmol/l for two consecutive weeks. Due to our ethical permits for animal experiments (see above), mice that were considered diabetic according to these criteria had to be euthanized. Pancreas tissue samples were frozen in isopentane and cooled with liquid nitrogen, or alternatively, fixed and embedded in paraffin. Tissue sections (5–6 μm) were stained with hematoxylin and eosin (H&E) to assess insulitis. The sections were evaluated randomly and blinded by microscopy. Insulitis was scored in at least 40 non-overlapping islets per mouse using the following grading: no infiltration or intact islets (score 0), peri-insulitis (score 1), less than 50% of islets infiltrated (score 2), and more than 50% of islets infiltrated (score 3). The insulitis index was calculated according to the following formula [62]: Insulitis index = (0 × n_0_) + (1 × n_1_) + (2 × n_2_) + (3 × n_3_) / 3 (n_0_ + n_1_ + n_2_ + n_3_)

Where n_0_-n_3_ denotes the number of islets of scores 0–3.

### Immunohistochemistry

Consecutive 8 μm cryosections were prepared from 5 different levels of the pancreas and stained with optimal concentration of anti-CD4, anti-CD8 (both from Affymetrix; Santa Clara, Ca, USA), anti F4/80 and anti-FoxP3 (eBioscience, Nordic Biosite, Täby, Sweden). The slides were then incubated with polymer horseradish peroxidase-labeled secondary antibodies, and 3,3-Diaminobenzidine (DAB), respectively. The stained sections were analyzed in a Leica DMRX microscope. At least 40 islets were analyzed from each pancreas. The scoring of the extent of staining was performed as follows:

CD4/CD8: score 1, cells located peripherally, encircling the islet; score 2, cells infiltrating up to 1/3 of the islet; score 3, cells infiltrating 1/3 to 2/3 of the islet; score 4, cells infiltrating more than 2/3 of the islet.

F4/80: score 1, cells located peripherally, encircling the islet; score 2, discrete presence of cells in islet; score 3, moderate presence of cells in islet; score 4, marked presence of cells in islet.

FoxP3: score 1, only a few positive cells; score 2, low density of positive cells in islet; score 3, moderate density of cells in islet; score 4, high density of cells in islet.

### Antibodies and flow cytometry

Single cell suspensions were prepared from spleens and pancreatic lymph nodes by disaggregation through 70 μm filters. The number of cells obtained from spleens and pancreatic lymph nodes were determined by flow cytometry by including AccuCount beads (Sphereotech, Lake Forest, IL) in the antibody-stained cell suspensions. The following antibodies were purchased from Biolegend (Nordic Biosite, Täby, Sweden): CD11b-Alexa700, Ly6G-APC-Cy7, F4/80-PE-Cy7, streptavidin Brilliant Violet 605. The following antibodies were purchased from BD Biosciences (San Jose, Ca, USA): CD19-PerCP-Cy5.5, Ly6C-biotin, SiglecF-PE. Prior to surface staining, cells were incubated with the 2.4G2 (anti-CD16/CD32) antibody to prevent unspecific binding. Cells were then stained with the above-mentioned antibodies in FACS buffer (PBS supplemented with 5% fetal calf serum and 0.05% NaN_3_ (Sigma-Aldrich, St. Louis, MO). Fixable viability dye e-Fluor506 (eBioscience) was used for detecting and excluding dead cells from the analyses. Cells were analyzed using an LSRII flow cytometer (BD Biosciences) and FlowJo software (Tree Star, Ashland, OR).

### Statistical analyses

Results are presented as mean and standard error of the mean (SEM). Differences between two groups were considered significant when *P* <0.05 as assessed by the non-parametric Mann-Whitney U test. Differences in disease incidence were assessed by Mantel-Cox log-rank test analysis. Statistical analysis was performed using the GraphPad Prism 6 software (GraphPad Software, San Diego, CA).

## Results

### Paquinimod treatment prevents development of diabetes in the NOD mouse

**Fig 1:**
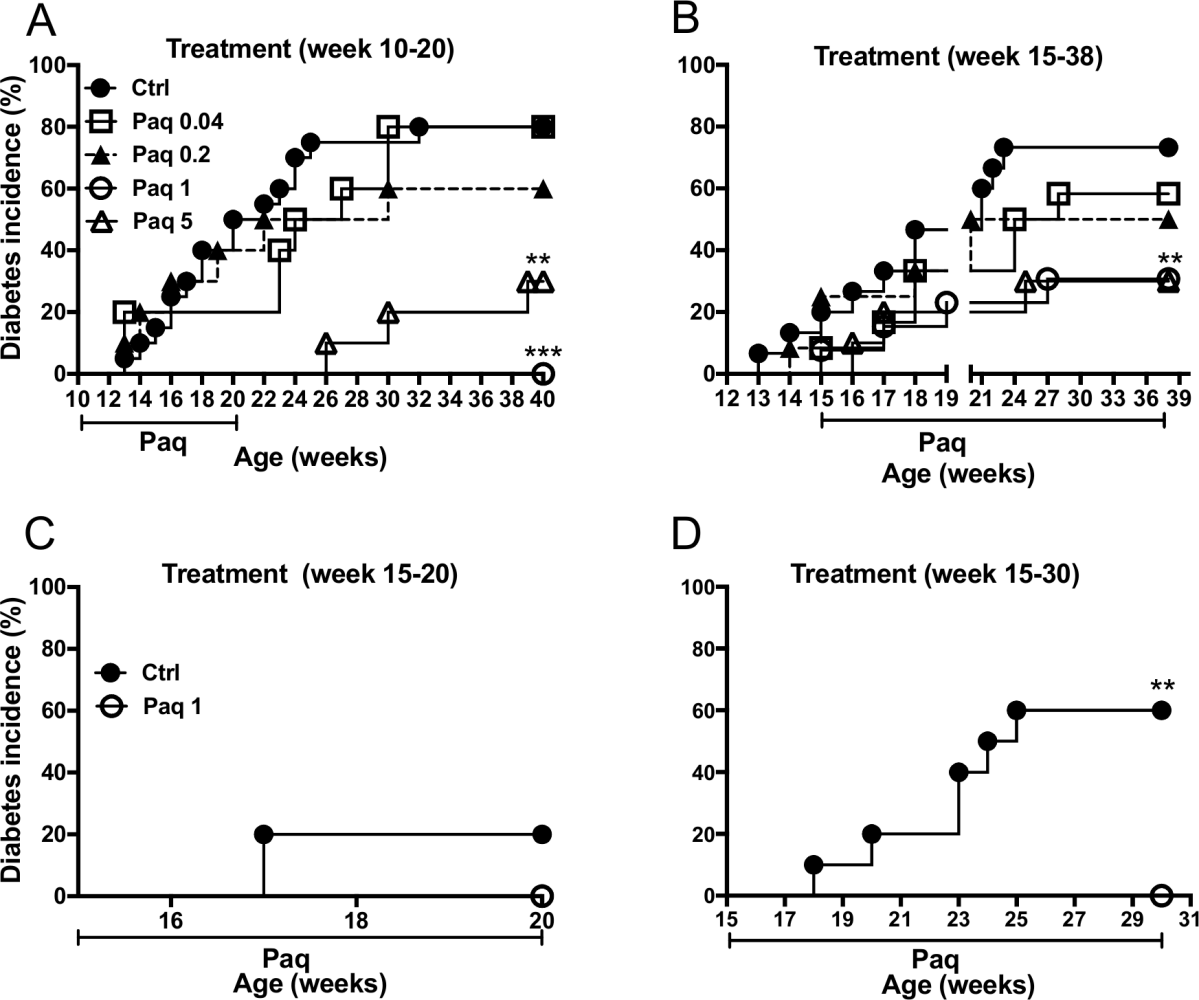
Delayed onset and reduced susceptibility to diabetes in paquinimod-treated NOD mice. Incidence of diabetes in mice treated with different doses of paquinimod (mg/kg/day; n = 10 for each dose) or vehicle (Ctrl; n = 20) from 10 to 20w of age A) or 15 to 38 w of age B). In the experiment in C) and D) NOD mice were treated with 1mg/kg/day of paquinimod or vehicle starting at 15w of age and two groups of mice (treated n = 10; controls n = 10) were sacrificed after 5 weeks of treatment (20w of age) C), and two additional groups (treated n = 10; controls n = 10) were sacrificed after 15 weeks of treatment (30w of age). Incidence of diabetes in treated groups compared to the control group (**, *p <* 0.01, ***, *p <* 0.001, by Mann Whitney U test).

To assess the preventive efficacy of paquinimod on diabetes development in female NOD mice, we treated groups of mice with daily doses of 0.04, 0.2, 1, and 5 mg/kg/day of paquinimod from week 10 of age until week 20 of age. Glycosuria was analyzed on a weekly basis from 10 weeks of age until the endpoint of the experiment at 40 weeks of age. As shown in **Fig 1A**, there is a clear dose-dependent reduction in diabetes development in the paquinimod-treated mice.

In the control group 80% of the mice (16 out of 20) developed diabetes. The incidence of diabetes was the same in the group that received 0.04 mg/kg/day of paquinimod (8 out of 10 mice, 80%), whereas 60% of the mice (6 out of 10) that received 0.2 mg/kg/day of paquinimod developed diabetes. None of the mice treated with 1 mg/kg/day of paquinimod developed diabetes (p<0.001), while the incidence of disease development was 30% (3 out of 10; p<0.01) in the group of mice that received 5mg/kg/day of paquinimod. The calculated average week of diabetes onset was also significantly delayed in the groups of mice treated with 1 and 5 mg/kg/day of paquinimod as compared to untreated controls (p<0.0001 and p<0.001, respectively) (**S1 Table**).

The time of onset of diabetes in NOD mice is variable, while development of insulitis is more homogenous such that at 15 weeks of age the NOD mouse generally displays extensive leukocyte infiltration of the pancreatic islets [3–5]. To elucidate the efficacy of paquinimod on disease development in NOD mice with extensive insulitis, we treated groups of mice with the same doses of paquinimod as was used in the experiment in **Fig 1A**, but starting the treatment at 15 weeks of age and continued until the endpoint at 38 weeks of age. A dose-dependent reduction of diabetes incidence can also be seen in in this experiment (**Fig 1B**). As summarized in **S1 Table**, the incidence of diabetes in the groups treated with 1 and 5mg/kg/day of paquinimod was 30% (3 out of 10 mice) and 31% (4 out of 13 mice), respectively and the incidence is significantly reduced in both groups as compared to the untreated control group (73.3%; 11out of 15 mice) (p<0.01). The week of diabetes onset is significantly reduced in these two groups (p<0.05). There is also a trend towards lower incidence of diabetes (50%; 6 out of 12 mice) and (58.3%; 7 out of 12 of mice) as compared to the control group in the groups of NOD mice treated with lower doses of paquinimod (0.2 and 0.04mg/kg/day, respectively) (**S1 Table**).

### Paquinimod treatment prevents progression of insulitis in the NOD mouse

Insulitis leads to destruction of β-cells and causes the development of T1D [3]. To elucidate a possible ameliorating effect of paquinimod treatment on insulitis, we treated 15 weeks old NOD mice with a dose of 1 mg/kg/day, which in the experiments described above showed similar efficacy as the 5mg/kg/day dose on the inhibition of diabetes development. One group of mice was sacrificed at the beginning of the experiment to serve as baseline for the histological analyses. Further, both at the age of 20 weeks and 30 weeks, respectively, one control group (n = 10) and one treated group (n = 10) of mice were sacrificed. As can be seen in **Fig 1C**, while all of the mice in the paquinimod-treated group scored negative at 20 weeks of age, a fraction of the mice in the control group had developed diabetes. Similarly, at 30 weeks of age all of the mice in the paquinimod-treated group still scored negative (**Fig 1D**). As would be expected, however, at this later time point an increased fraction of mice had developed diabetes in the control group. The incidence of diabetes was significantly reduced and the week of onset significantly delayed in the paquinmod-treated group analyzed at 30 weeks of age (**S2 Table)**.

**Fig 2:**
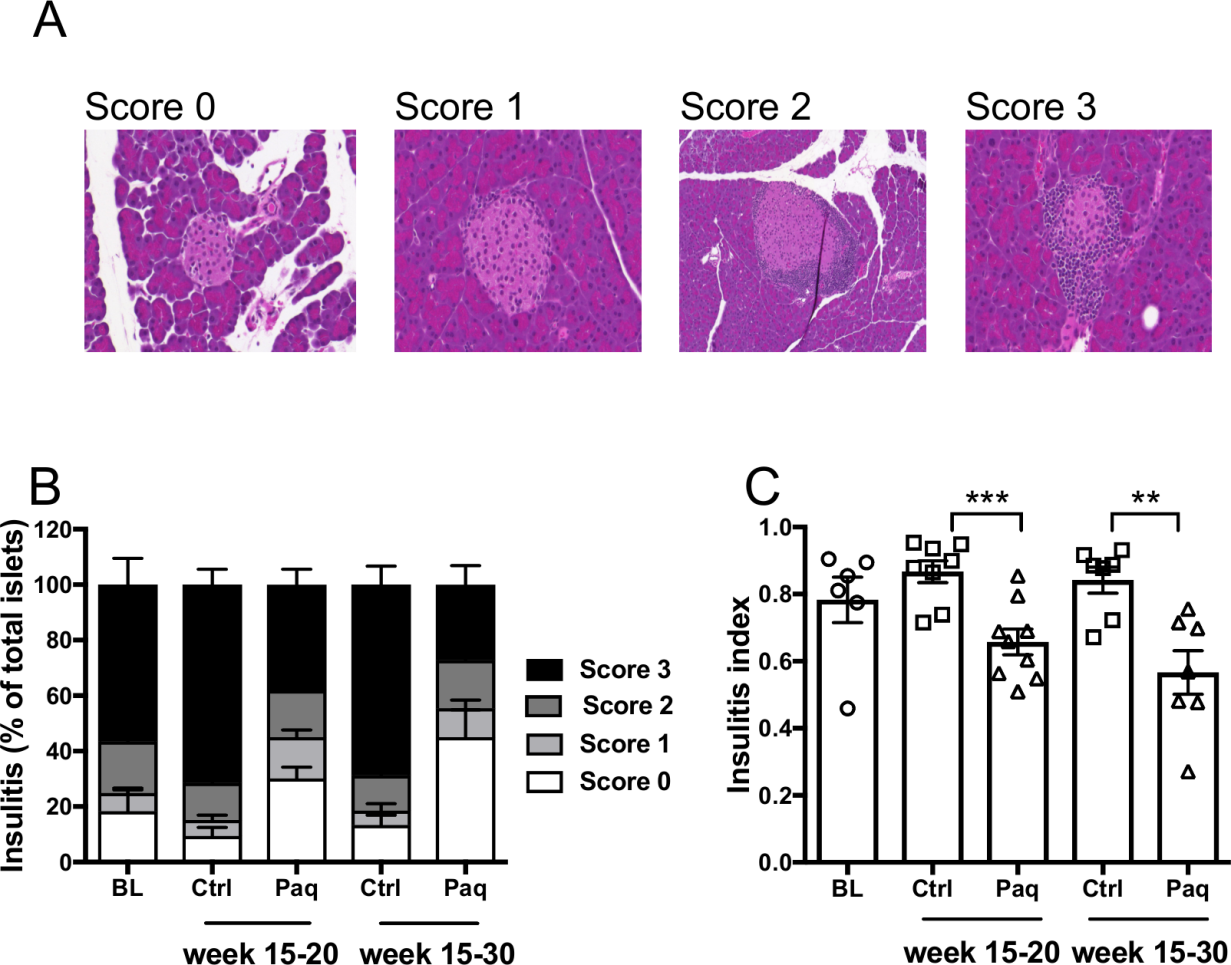
Reduced insulitis in paquinimod treated NOD mice. Cohorts of 15 w old mice were treated either with paquinimod (Paq; 1 mg/kg/day) or vehicle (Ctrl) as indicated. Groups of mice were sacrificed at the start of the experiment (Baseline n = 6), at 20 w of age (Ctrl n = 8; Paq n = 9) and at 30w of age (Ctrl n = 7; Paq n = 7). Serial sections of pancreatic tissue were prepared, stained with H&E and analyzed microscopically. A) Representative images are shown; scale bar in image: 100 μm. Insulitis scores B), and insulitis indexes C) are shown. In B), the extent of mononuclear cell infiltration was scored from 0 through 3. Score 0 (open bars), score 1 (light grey bars), score 2 (medium grey bars), score 3 (black bars). In C) insulitis index was calculated as described in *Materials and Methods*. Insulitis was scored by examining a minimum of 40 islets per animal. **, *p* < 0.01, ***, *p* < 0.001 by Mann Whitney U test.

Next, pancreatic tissue sections of NOD mice from both control and paquinimod-treated groups were histologically assessed (**Fig 2A**). As summarized in **Fig 2B** and **S3 Table**, paquinimod-treated animals displayed reduced level of infiltration of pancreatic islets as compared to untreated controls.

Even though infiltrated islets could also be detected in paquinimod-treated NOD mice, the frequency of non-infiltrated islets (score 0) was significantly higher in the treated group compared to the control group. Thus, the mice that were treated with paquinimod for 5 weeks (*n = 9*), had 30.1% of intact islets as compared to 9.5% in controls (*n = 8*) (*p* <0.01). Further supporting a beneficial effect of paquinimod treatment on insulitis, the frequency of score 3 islets was found to be significantly higher in controls, with 71.4% of islets having more than 50% of infiltration as opposed to 38% in treated mice (*p* <0.01). Similarly, in the group of mice that was treated with paquinimod for 15 weeks, the pancreata had 45% intact islets, while only 13.3% islets were intact in control mice (*p* <0.05). Furthermore, the treated mice in that group displayed reduced frequency of score 3 islets (27.2%, *n = 7*) compared to untreated controls (68.6%, *n = 7*) (*p* <0.01). Importantly, even though the 15 weeks old mice as shown in **Fig 2B** display extensive insulitis already at the start of the experiment, we found less pronounced insulitis in the mice treated for 5 and 15 weeks with paquinimod compared to the age-matched untreated controls. These results indicate that the treatment may prevent progression of already established insulitis (**S3 Table**).

The histological analyses also revealed that the mean insulitis index for treated mice was significantly lower after 5 weeks of treatment (0.7 ± 0.1, *n = 9*), vs control (0.8 ± 0.0, *n = 9*) (p<0.001) (**Fig 2C**). Moreover, this significant trend was preserved even after 15 weeks of treatment, as the insulitis index for treated group (*n = 7*) was 0.6 ± 0.1 (SEM), and 0.9 ± 0.1 (SEM) for the control group (*n = 7*) (p<0.01).

We also analyzed pancreatic tissue sections prepared from three paquinimod-treated (1mg/kg/day) and three control mice in the experiment shown in (**Fig 1B**) for the expression of different cellular markers related to disease pathogenesis (**S1A Fig**). There was also reduced, even though not significant, extent of infiltration by CD4- and CD8-positive cells in paquinimod-treated mice (**S1B Fig)**. Notably, there was a trend towards reduced frequency of islets heavily infiltrated by CD4- and CD8-positive cells **(S1C Fig)**. In contrast, there were only minor effects on the extent of infiltration by F4/80^+^ macrophages and Foxp3^+^ regulatory T cells.

### Paquinimod treatment reduces the frequencies of F4/80^+^ macrophages and Ly6C^hi^ monocytes in the splenic myeloid cell population of NOD mice

**Fig 3:**
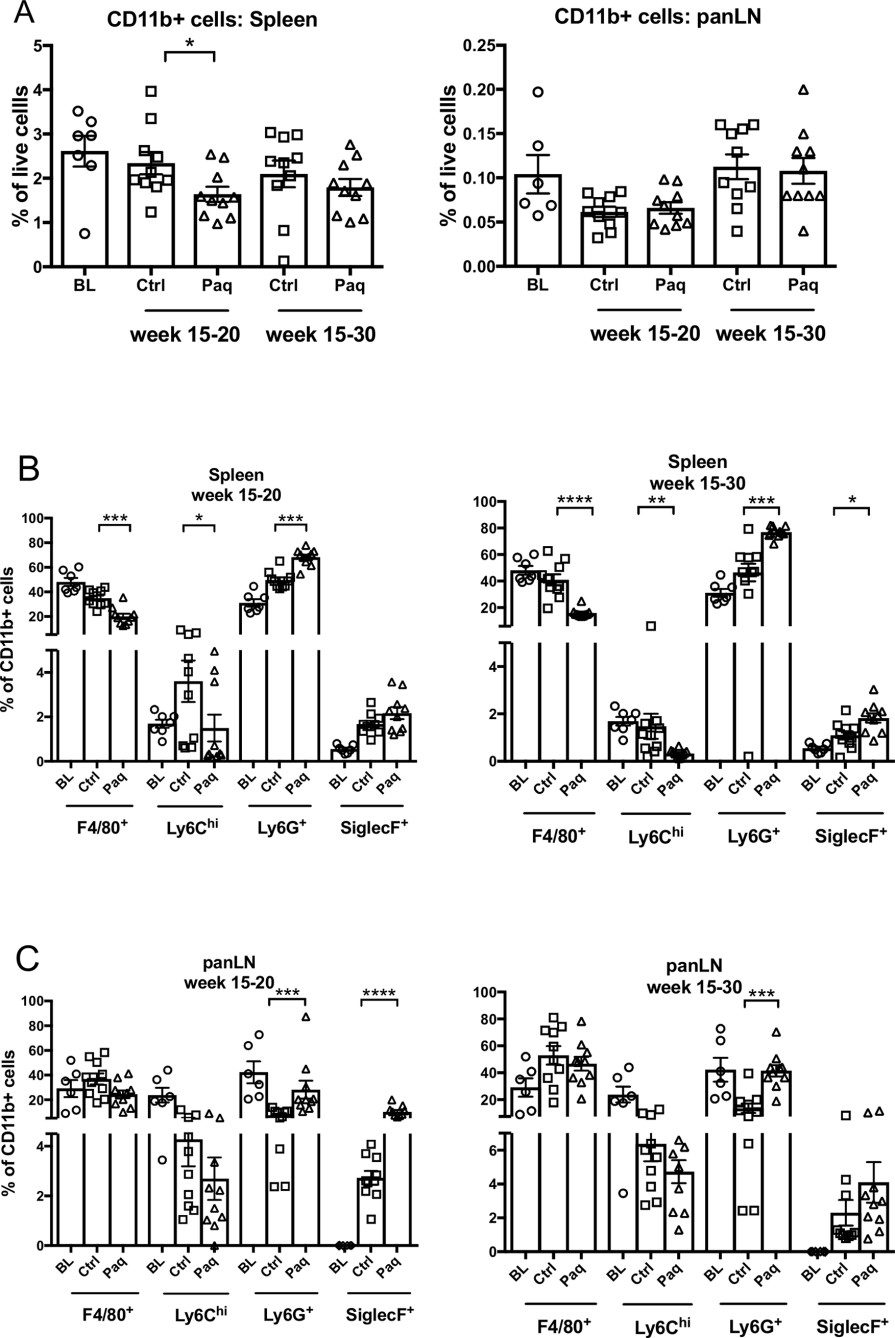
Paquinimod treatment reduces the frequency of Ly6C^hi^ and F4/80^+^ cells in spleen of NOD mice. Cells from spleen and panLN of the mice in Fig 1C and 1D were analyzed by flowcytometry. A), percentage of single CD19^-^ CD11b^+^ cells out of total viable cells, as well as percentage of Ly6C^hi^ inflammatory monocytes, Ly6G^+^ neutrophils, and SiglecF^+^ eosinophils among total CD11b^+^ cell population is shown both for B) spleens and C) pancreatic lymph nodes of mice. *p < 0.05, **p < 0.01, ***p < 0.001, ****p < 0.0001, Mann–Whitney U test.

Previous studies from several laboratories have demonstrated the efficacy of Q compounds in various disease models. Some of those studies suggested that the Q compounds might ameliorate inflammatory disease by affecting myeloid cell populations such as DCs and monocytes [43, 54, 56, 57]. We therefore used flow cytometry to investigate possible effects of paquinimod on subpopulations of CD11b^+^ cells, both in spleen and pancreatic lymph nodes (panLN) using the gating strategy shown in **S2A Fig**. The frequency (**Fig 3A**) and the absolute number (**S2B Fig**) of splenic CD11b^+^ cells was significantly reduced after 5 weeks of treatment with paquinimod, and although not significant, there was a trend towards reduction of these cells after 15 weeks of treatment. However, there was no effect of paquinimod treatment on CD11b^+^ cells in panLNs (**Fig 3A**).

The frequency (**Fig 3B)** and absolute number (**S2B Fig**) of F4/80^+^ macrophages and Ly6C^hi^ inflammatory monocytes in spleen was significantly reduced both after 5 and 15 weeks Paquinimod treatment. The decrease in frequency of these populations resulted in a corresponding increase in the frequency of Ly6G^+^ neutrophils and SiglecF^+^ eosinophils (**Fig 3B)**, but not in absolute numbers (**S2B Fig**). Although the frequency of F4/80^+^ macrophages in panLN was not changed in the paquinimod-treated mice, a trend towards a decrease in Ly6C^hi^ inflammatory monocytes could be seen. Similar to the effect of paquinimod in spleen, there was an increase in the frequency on Ly6G^+^ neutrophils and SiglecF^+^ eosinophils in panLN of treated mice (**Fig 3C**).

## Discussion

The aim of this study was to investigate whether treatment of NOD mice with the immunomodulatory Q compound paquinimod would show efficacy on the development of diabetes. We first treated of 10 weeks old NOD mice with various doses of paquinimod until the mice were 20 weeks of age. This is an age at which extensive insulitis is observed in most pancreatic islets and some of the mice will have developed diabetes. Treating the mice using this preventive protocol was in a dose-dependent way sufficient not only to significantly reduce the incidence of diabetes but also to significantly delay the onset of the disease. These results suggested to us that the 10 weeks of treatment had delayed the progression of insulitis, thereby delaying both the week of onset and the overall incidence of diabetes.

To investigate the efficacy of paquinimod on development of diabetes in mice with more progressed insulitis, we initiated treatment in 15 weeks old mice. At this age, insulitis would have progressed such that most of the islets of Langerhans would be heavily infiltrated by leukocytes and thereby causing extensive cell death of the insulin producing β-cells. The same doses of paquinimod that showed efficacy in the preventive treatment protocol also significantly reduced both the onset of and the incidence of diabetes in the mice that were treated from 15 to 40 weeks of age. These results provided further support for the hypothesis that paquinimod treatment delays the progression of insulitis. Only a few of the mice that entered the experiment were diabetic and hence we could not draw firm conclusions on putative therapeutic effects of paquinimod on established diabetes. Because of local legislation for work with laboratory animals, we cannot keep diabetic mice for more than one week. Hence, we could not formally address this therapeutic effect experimentally.

We obtained direct experimental support for a therapeutic effect of paquinimod treatment on insultis by scoring pancreatic tissue sections, that were prepared from mice treated with paquinimod from week 15 of age to either week 20 or week 30 of age. The results obtained from the analyses of both groups of mice were very similar. In the pancreata of the treated mice, the proportion of heavily infiltrated islets was significantly reduced and the proportion of non-infiltrated islets was significantly increased as compared to tissue sections derived from untreated controls. We conclude from these experiments that paquinimod treatment delays the progression of insulitis. We further speculate that regeneration of pancreatic β-cell mass might explain the increased frequency of non-infiltrated islets found in the treated mice. Previous studies using various therapeutic regimens in NOD mice have reported on the regeneration of β-cell mass in the treated mice [63–66]. Proliferation of β-cells is a main mechanism for increasing β-cell mass in the mouse [67], but other mechanisms may also contribute [68, 69].

The frequency and absolute number of splenic CD11b^+^ myeloid cells and Ly6C^hi^ inflammatory monocytes was reduced in 15 weeks old NOD mice that had been treated with paquinimod until 20 and 30 weeks of age, respectively. This effect was selective since the absolute number of splenic SiglecF^+^ eosinophils and Ly6G^+^ neutrophils did not change. The effect of treatment on panLN inflammatory monocytes and eosinophils showed similar trends. Taken together, paqunimod-treated NOD mice displayed similar changes in splenic myeloid cell populations as previously observed in treatment studies in other disease models either using paquinimod or other structurally related Q compounds [55–58].

Depletion of macrophages and circulating monocytes in NOD mice was shown to delay both onset and incidence of diabetes [70] and importantly, also in an acute diabetes model involving transfer of diabetogenic T cells [71]. The latter result indicates that macrophages and monocyte are crucial for diabetes development even in the effector phase of the T cell response. Conversely, overexpression of CCL2 in NOD pancreatic β-cells that leads to recruitment of large numbers of monocytes to the pancreas caused insulitis [72] and even the development of diabetes [73]. Taken together, these previous studies indicate that both macrophages and monocytes may be important for development of diabetes in the NOD mouse. The spleen has been shown to be a reservoir for monocytes during inflammatory conditions [74]. Thus, the reduction of the number of splenic Ly6C^hi^ inflammatory monocytes caused by paquinimod treatment in the experiments reported in here, might potentially contribute to the observed ameliorating effects on diabetes.

Our previous studies indicated that paquinimod, as well as the structurally related Q compound tasquinimod, interfere with the accumulation of inflammatory monocytes and eosinophils in the inflamed peritoneum [42] and in tumor tissue [54], respectively. Along these lines, reports from other laboratories showed that other structurally related Q compounds had similar effects. Thus, laquinimod reduced the transmigration of LPS-stimulated monocytes in an in vitro model [75] and linomide prevented leukocyte-endothelium interactions and extravasation in a rat model of TNFα-induced hepatic injury [76]. Finally, in a recent report paquinimod was shown to increase the rolling velocity of leukocytes on inflamed endothelium in vivo [77]. Collectively, these previous studies suggest that recruitment of leukocytes to sites of inflammation might be a common mode of action of paquinimod and other structurally related Q compounds, that may explain their efficacy in various models of inflammation. In the present report, we treated NOD mice with paquinimod from the age of 10 or 15 weeks of age. At 8 weeks of age a new set of “late myeloid” genes are expressed in pancreatic islets of the NOD mouse [78], indicative of arrival of new myeloid cells such as monocytes. We speculate that paqunimod might interfere with the recruitment of such late arriving myeloid cells, thereby interfering with the progression of insulitis.

It well established that development of T1D in the NOD mouse is T cell dependent [3, 10, 11]. In the present study, we also analyzed sections of pancreatic tissue from control and paquinimod-treated mice using immuno-histochemistry. The islets of pancreas from treated mice displayed a trend towards reduced frequency of islets that were heavily infiltrated by CD4 and CD8 T cells. Because only a few mice were analyzed this reduction did not reach significance and therefore we cannot draw firm conclusions from these analyses. Nevertheless, this reduction probably reflects the overall reduced insulitis in the paquinimod-treated mice. Further, studies from several laboratories have suggested that myeloid cells might have a functional impact on the T cell response in mice treated with Q compounds. Thus, our laboratory previously reported that paquinimod treatment ameliorated EAE, that is a T cell dependent murine model of multiple sclerosis. The amelioration correlated with reduced number of splenic Ly6C^hi^ inflammatory monocytes and we could show that these cells are important for development of EAE [43]. Similar amelioration of disease in the EAE model was observed in mice treated with the Q compound laquinimod [56, 57]. Modulation of the myeloid cell compartment, either monocytes or myeloid DCs, was also reported in those studies and it was proposed that the modulation might reduce the frequency of disease-causing effector T cells [56, 57]. We therefore speculate, that also in the NOD mouse model the effect of paquinimod on myeloid cells might lead to a modulation of T cell function. In support of this view, Weiss et al reported on a shift from Th1 to Th2 type cytokine response in spleen cells from NOD mice treated with the Q compound linomide [79].

Although we acknowledge the limitations of the NOD model for predicting the possible effects of the same compound in human disease, the spontaneous nature of disease development in the NOD mouse has made it the most used pre-clinical model of human T1D. Many of the previous reports on the efficacy of Q compounds in inflammatory disease and cancer have involved the use of models in which disease is induced and not spontaneously developing as in the NOD mouse. The demonstration of the long-term protection from development of overt diabetes in NOD mice treated with paquinimod provides an attractive non-cytotoxic basis for clinical trials in type1diabetes. Phase I clinical trials in patients with systemic lupus erythematosus [41] and systemic sclerosis (unpublished results) have been performed and demonstrated good tolerability for this compound.

## Supporting information

S1 Table Average week of onset, incidence of diabetes and survival of control and paquinimod-treated NOD mice. Average week of disease onset was calculated until ^a^ week 40 and ^b^week 38 for the therapeutic treatment groups. For the mice that had not developed diabetes at the endpoints at ^a^week 40 or ^b^week 38, respectively those weeks were considered as the week of onset. Data are presented as mean ± SEM. Statistical significance compared to control group (Ctrl) was calculated by Mann Whitney U test for the onset data, and by the log-rank test for the incidence and survival data (*, *p* <0.05;**, *p <* 0.01; ***, *p <* 0.001; ****p <0.0001).
(PDF)

S2 Table Delayed onset and reduced incidence of diabetes in paquinimod-treated NOD mice. ^a^ Average onset week was calculated for these mice until ^a^week 20 or ^b^week 30. For the mice that did not develop diabetes the onset week was considered as week 20 or week 30, respectively. Data presented as mean ± SEM. Statistically significant (*, *p <* 0.05) compared to control group (Ctrl) by Mann Whitney U test for the onset data, and by the log-rank test for the incidence and survival data. Incidence of treated mice compared to control group (*, *p <* 0.05, **, *p <* 0.01).
(DOCX)

S3 Table Reduced severity of insulitis in paquinimod-treated NOD mice. Average score was calculated from histological analyses of islet infiltration in pancreata isolated from mice at indicated weeks of sacrifice or, alternatively, isolated from mice that were sacrificed when proved to be diabetic. Data are presented as the mean percentage of islets with scores 0–3 within each of the indicated groups of mice ± SEM. Statistically significant (*, *p <* 0.05, **, *p <* 0.01) by Mann Whitney U test for each score as compared to control (Ctrl) group.
(DOCX)

S1 Fig Reduced frequency of heavily T cell-infiltrated pancreatic islets in paquinimod-treated NOD mice. Groups of mice were treated either with paquinimod (Paq; 1 mg/kg/day, n = 3) or vehicle (Ctrl, n = 3) from 15 w– 38 w of age. Serial sections of pancreatic tissue were prepared, stained with H&E and with various antibodies and analyzed microscopically. A) Representative images of CD4, CD8, F4/80 and FoxP3 staining in consecutive tissue sections of the same pancreatic islet are shown (Scale bar: 100 μm). B) Mean scores of indicated markers in pancreatic islets, calculated as described in *Materials and Methods*. C) Percentage of Scores 1 through 4 for each marker in ctrl and paq-treated mice. Score 0 (open bars), score 1 (light grey bars), score 2 (medium grey bars), score 3 (striped bars), score 4 (black bars). A minimum of 40 islets was examined for each animal.
(TIF)

S2 Fig A), gating strategy for the identification of F4/80^+^, Ly6C^hi^, Ly6G^+^, SSC^hi^ SiglecF^+^ cells within the CD19^-^ CD11b^+^ cell population in spleen and panLN, that are shown in **Fig 3**. B) Absolute number of splenic myeloid cell population shown in **Fig 3B**.
(TIF)
