# Supplementary material for: Paquinimod prevents development of diabetes in the non-obese diabetic (NOD) mouse
Source: PLoS One. 2018 May 9;13(5):e0196598. doi: 10.1371/journal.pone.0196598 (PMC5942776; doi:10.1371/journal.pone.0196598)
Supplement: S1 Table — Average week of disease onset was calculated until a week 40 and bweek 38 for the therapeutic treatment groups. For the mice that had not developed diabetes at the endpoints at aweek 40 or bweek 38, respectively those weeks were considered as the week of onset. Data are presented as mean ± SEM. Statistical significance compared to control group (Ctrl) was calculated by Mann Whitney U test for the onset data, and by the log-rank test for the incidence and survival data (*, p <0.05;**, p < 0.01; ***, p < 0.001; ****p <0.0001). (PDF) [file pone.0196598.s001.pdf]

S1 Table. Delayed onset and reduced incidence of diabetes in paquinimod-treated NOD mice

Treatment w10 to w20	n	Onset (week)[a]	Incidence (%)	Survival (%)
Ctrl	20	23.8 ± 2.1	80	20
0.04 mg/kg/day	10	26.3 ± 2.9	80	20
0.2 mg/kg/day	10	27.4 ± 3.7	60	40
1 mg/kg/day	10	40.0 ± 0.0****	0***	100
5 mg/kg/day	10	37.5 ± 1.6***	30**	70

Treatment w15 to w38	n	Onset (week)[b]	Incidence (%)	Survival (%)
Ctrl	15	23.3 ± 2.5	73.3	26.7
0.04 mg/kg/day	12	27.2 ± 2.9	58.3	41.7
0.2 mg/kg/day	12	26.5 ± 3.3	50	50
1 mg/kg/day	13	32.3 ± 2.6*	31**	69
5 mg/kg/day	10	32.4 ± 2.9*	30**	70
