# Supplementary material for: Paquinimod prevents development of diabetes in the non-obese diabetic (NOD) mouse
Source: PLoS One. 2018 May 9;13(5):e0196598. doi: 10.1371/journal.pone.0196598 (PMC5942776; doi:10.1371/journal.pone.0196598)
Supplement: S2 Table — a Average onset week was calculated for these mice until aweek 20 or bweek 30. For the mice that did not develop diabetes the onset week was considered as week 20 or week 30, respectively. Data presented as mean ± SEM. Statistically significant (*, p < 0.05) compared to control group (Ctrl) by Mann Whitney U test for the onset data, and by the log-rank test for the incidence and survival data. Incidence of treated mice compared to control group (*, p < 0.05, **, p < 0.01). (DOCX) [file pone.0196598.s002.docx]

**S2 Table.** Delayed onset and reduced incidence of diabetes in paquinimod-treated NOD mice

| Group | n | Onset (week) | Incidence (%) | Survival (%) |
| --- | --- | --- | --- | --- |
| Ctrl (week 15-20) | 10 | 18.8 ± 0.7^a^ | 20 | 80 |
| 1 mg/kg/day (week 15-20) | 10 | 20.0 ± 0.0 | 0 | 100 |
| Ctrl (week 15-30) | 10 | 25.3 ± 1.4^b^ | 60 | 40 |
| 1 mg/kg/day (week 15-30) | 10 | 30.0 ± 0.0* | 0** | 100 |
