# Supplementary material for: Paquinimod prevents development of diabetes in the non-obese diabetic (NOD) mouse
Source: PLoS One. 2018 May 9;13(5):e0196598. doi: 10.1371/journal.pone.0196598 (PMC5942776; doi:10.1371/journal.pone.0196598)
Supplement: S3 Table — Average score was calculated from histological analyses of islet infiltration in pancreata isolated from mice at indicated weeks of sacrifice or, alternatively, isolated from mice that were sacrificed when proved to be diabetic. Data are presented as the mean percentage of islets with scores 0–3 within each of the indicated groups of mice ± SEM. Statistically significant (*, p < 0.05, **, p < 0.01) by Mann Whitney U test for each score as compared to control (Ctrl) group. (DOCX) [file pone.0196598.s003.docx]

**S3 Table.** Reduced severity of insulitis in paquinimod-treated NOD mice

| Group | n | Score 0 | Score 1 | Score 2 | Score 3 |
| --- | --- | --- | --- | --- | --- |
| Baseline | 6 | 18.3 ± 8.3 | 6.6 ± 1.2 | 18.6 ± 2.2 | 56.4 ± 9.5 |
| Ctrl (w 15-20) | 8 | 9.5 ± 3.1 | 5.7 ± 1.8 | 13.4 ± 1.8 | 71.4 ± 5.6 |
| Paq (w 15-20) | 9 | 30.2 ± 4.1** | 14.8 ± 2.6* | 16.9 ± 2.4 | 38.0± 5.5** |
| Ctrl (w 15-30) | 7 | 13.3 ± 3.6 | 5.2 ± 2.5 | 12.8 ± 3.5 | 68.6 ± 6.7 |
| Paq (w 15-30) | 7 | 45.0 ± 9.9* | 10.5 ± 3.0 | 17.3 ± 3.5 | 27.2 ± 6.9** |
