# Supplementary figures and images for: Paquinimod prevents development of diabetes in the non-obese diabetic (NOD) mouse
Source: PLoS One. 2018 May 9;13(5):e0196598. doi: 10.1371/journal.pone.0196598 (PMC5942776; doi:10.1371/journal.pone.0196598)

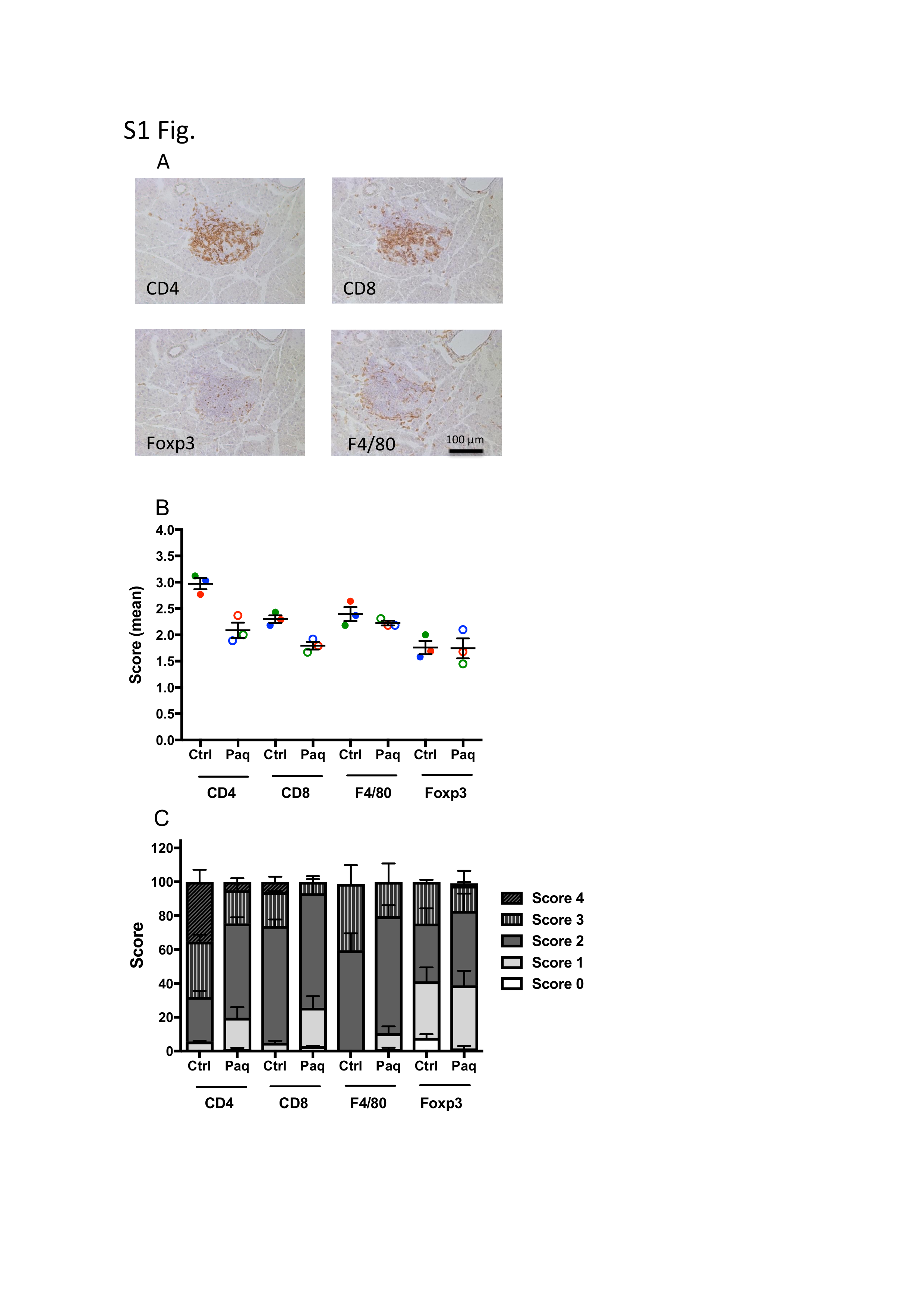

Supplement: S1 Fig — Groups of mice were treated either with paquinimod (Paq; 1 mg/kg/day, n = 3) or vehicle (Ctrl, n = 3) from 15 w– 38 w of age. Serial sections of pancreatic tissue were prepared, stained with H&E and with various antibodies and analyzed microscopically. A) Representative images of CD4, CD8, F4/80 and FoxP3 staining in consecutive tissue sections of the same pancreatic islet are shown (Scale bar: 100 μm). B) Mean scores of indicated markers in pancreatic islets, calculated as described in Materials and Methods. C) Percentage of Scores 1 through 4 for each marker in ctrl and paq-treated mice. Score 0 (open bars), score 1 (light grey bars), score 2 (medium grey bars), score 3 (striped bars), score 4 (black bars). A minimum of 40 islets was examined for each animal. (TIF) [file pone.0196598.s004.tif]

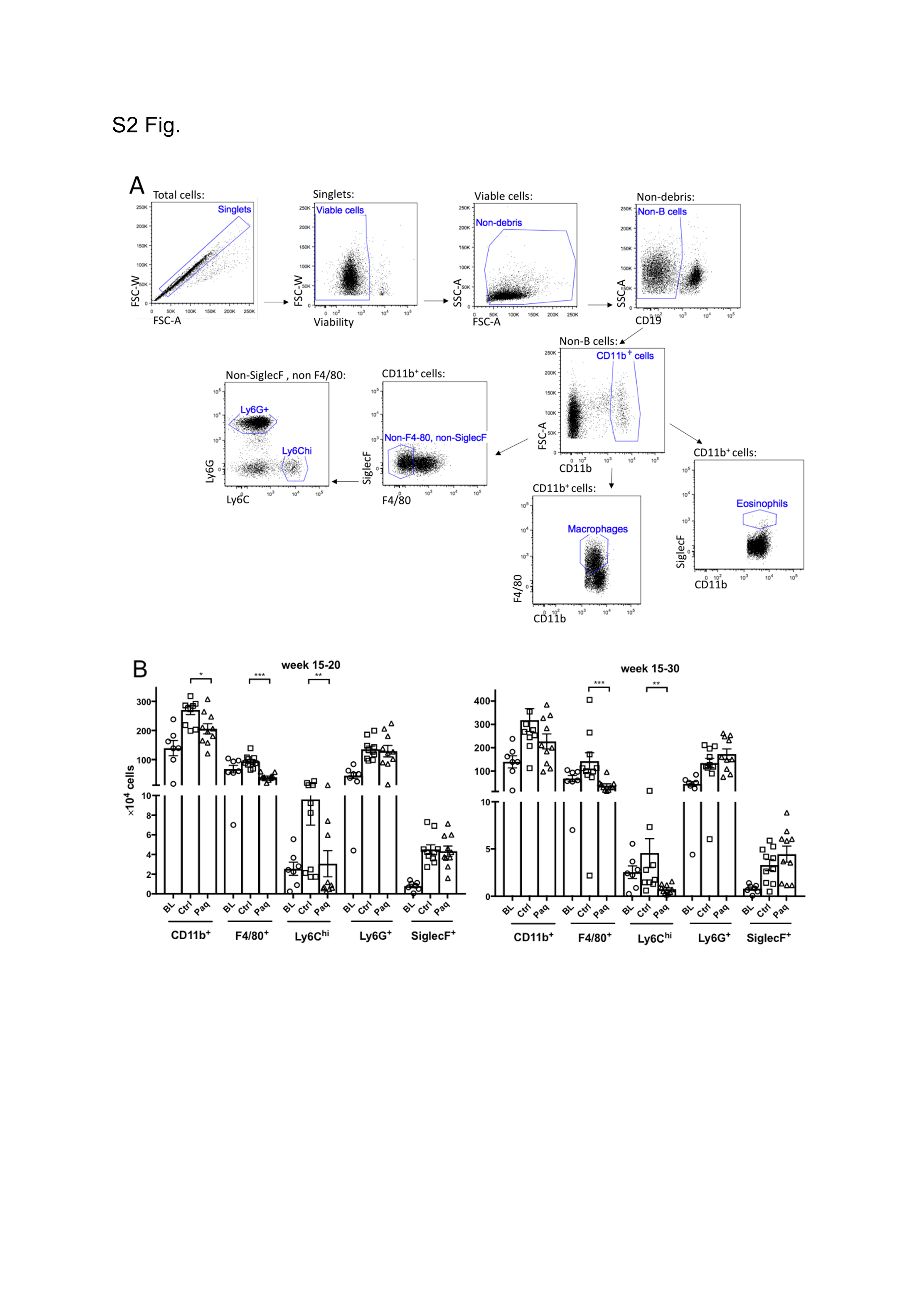

Supplement: S2 Fig — A), gating strategy for the identification of F4/80+, Ly6Chi, Ly6G+, SSChi SiglecF+ cells within the CD19- CD11b+ cell population in spleen and panLN, that are shown in Fig 3. B) Absolute number of splenic myeloid cell population shown in Fig 3B. (TIF) [file pone.0196598.s005.tif]
